# A novel echocardiographic hemodynamic index for predicting outcome of aortic stenosis patients following transcatheter aortic valve replacement

**DOI:** 10.1371/journal.pone.0195641

**Published:** 2018-04-26

**Authors:** Altayyeb Yousef, Benjamin Hibbert, Joshua Feder, Jordan Bernick, Juan Russo, Zachary MacDonald, Christopher Glover, Alexander Dick, Munir Boodhwani, Buu-Khanh Lam, Marc Ruel, Marino Labinaz, Ian G. Burwash

**Affiliations:** 1 Division of Cardiology, University of Ottawa Heart Institute, University of Ottawa, Ottawa, Ontario, Canada; 2 Division of Cardiac Surgery, University of Ottawa Heart Institute, University of Ottawa, Ottawa, Ontario, Canada; Scuola Superiore Sant'Anna, ITALY

## Abstract

**Objective:**

Transcatheter aortic valve replacement (TAVR) reduces left ventricular (LV) afterload and improves prognosis in aortic stenosis (AS) patients. However, LV afterload consists of both valvular and arterial loads, and the benefits of TAVR may be attenuated if the arterial load dominates. We proposed a new hemodynamic index, the Relative Valve Load (RVL), a ratio of mean gradient (MG) and valvuloarterial impedance (Zva), to describe the relative contribution of the valvular load to the global LV load, and examined whether RVL predicted patient outcome following TAVR.

**Methods:**

A total of 258 patients with symptomatic severe AS (indexed aortic valve area (AVA)<0.6cm^2^/m^2^, AR≤2+) underwent successful TAVR at the University of Ottawa Heart Institute and had clinical follow-up to 1-year post-TAVR. Pre-TAVR MG, AVA, percent stroke work loss (%SWL), Zva and RVL were measured by echocardiography. The primary endpoint was all cause mortality at 1-year post TAVR.

**Results:**

There were 53 deaths (20.5%) at 1-year. RVL≤7.95ml/m^2^ had a sensitivity of 60.4% and specificity of 75.1% for identifying all cause mortality at 1-year post-TAVR and provided better specificity than MG<40 mmHg, AVA>0.75cm^2^, %SWL≤25% and Zva>5mmHg/ml/m^2^ despite equivalent or better sensitivity. In multivariable Cox analysis, RVL≤7.95ml/m^2^ was an independent predictor of all cause mortality (HR 3.2, CI 1.8–5.9; p<0.0001). RVL≤7.95ml/m^2^ was predictive of all cause mortality in both low flow and normal flow severe AS.

**Conclusions:**

RVL is a strong predictor of all-cause mortality in severe AS patients undergoing TAVR. A pre-procedural RVL≤7.95ml/m^2^ identifies AS patients at increased risk of death despite TAVR and may assist with decision making on the benefits of TAVR.

## Introduction

Transcatheter aortic valve replacement (TAVR) has redefined the treatment strategy of patients with severe aortic stenosis (AS)[1],[2]. Randomized clinical trials have demonstrated that TAVR results in a dramatic improvement in survival in surgically inoperable severe AS patients, and an equivalent or better survival in intermediate and high risk surgical patients compared to surgical aortic valve replacement (AVR)[1,3,4]. While the majority of AS patients improve after TAVR, there remains a significant proportion of patients who fail to benefit[1,4]. Patients undergoing TAVR have a high prevalence of concomitant co-morbidities, which can reduce their life expectancy despite a successful TAVR procedure.

Confirmation of the presence of hemodynamically severe AS is essential to identify those patients most likely to benefit from TAVR. However, the standard hemodynamic indices used to determine AS severity, particularly mean transvalvular pressure gradient (MG) and aortic valve area (AVA), are highly dependent on the patient’s hemodynamics at the time of the diagnostic evaluation. MG and AVA are influenced by left ventricular (LV) function, transvalvular flow, and blood pressure, and can incorrectly reflect disease severity[5–7]. Alternative indices have been studied and proposed to evaluate AS severity [8–11]. Percent stroke work load loss (%SWL), the amount of energy lost ejecting blood across the stenotic aortic valve as a function of the total LV work produced, better predicted clinical outcome than MG and AVA in patients with asymptomatic AS[9]. Valvuloarterial impedance (Zva), a measure of the global left ventricular (LV) afterload, better predicted clinical outcome in patients with asymptomatic severe AS[8,11], as well as patients undergoing TAVR[12,13].

In AS patients, global LV afterload is a composite of valvular and arterial loads. The index Zva, although predicting patient prognosis, does not distinguish the magnitude of the loads attributable to the valvular or arterial components. Since TAVR only relieves valvular load, little benefit is likely to be realized in those AS patients with a high global LV afterload in which the vascular load dominates. In contrast, AS patients with a predominant valvular load would be expected to realize the greatest benefits following TAVR. Since MG provides a measure of valvular load and Zva of global LV load, the ratio of MG to Zva reflects the relative contribution of the valvular load as a function of global LV load. We hypothesized that this novel hemodynamic index, the ratio of MG to Zva, or “Relative Valve Load (RVL)” would predict the outcome of AS patients following TAVR and provide superior predictive value compared to conventional hemodynamic indices of AS severity. Patients with a larger RVL, in whom there is a relatively greater contribution of valvular load to the global LV load, would be expected to have the greatest benefits following TAVR.

## Method

### Patient population

A total of 303 patients underwent TAVR at the University of Ottawa Heart institute (UOHI) between February 2007 and October 2014. All patients considered for TAVR undergo comprehensive clinical, laboratory, echocardiographic and angiographic assessment. Each case is reviewed at a TAVR heart team rounds consisting of interventional and imaging cardiologists, cardiac surgeons, radiologists and a geriatrician. The decision to proceed with TAVR or surgical aortic valve replacement (SAVR) is based on the operative risk assessment and anatomic considerations. In patients undergoing TAVR, baseline demographic, clinical, echocardiographic, and angiographic data are collected in a dedicated TAVR database. Patients have clinical follow-up to one year.

**Fig 1:**
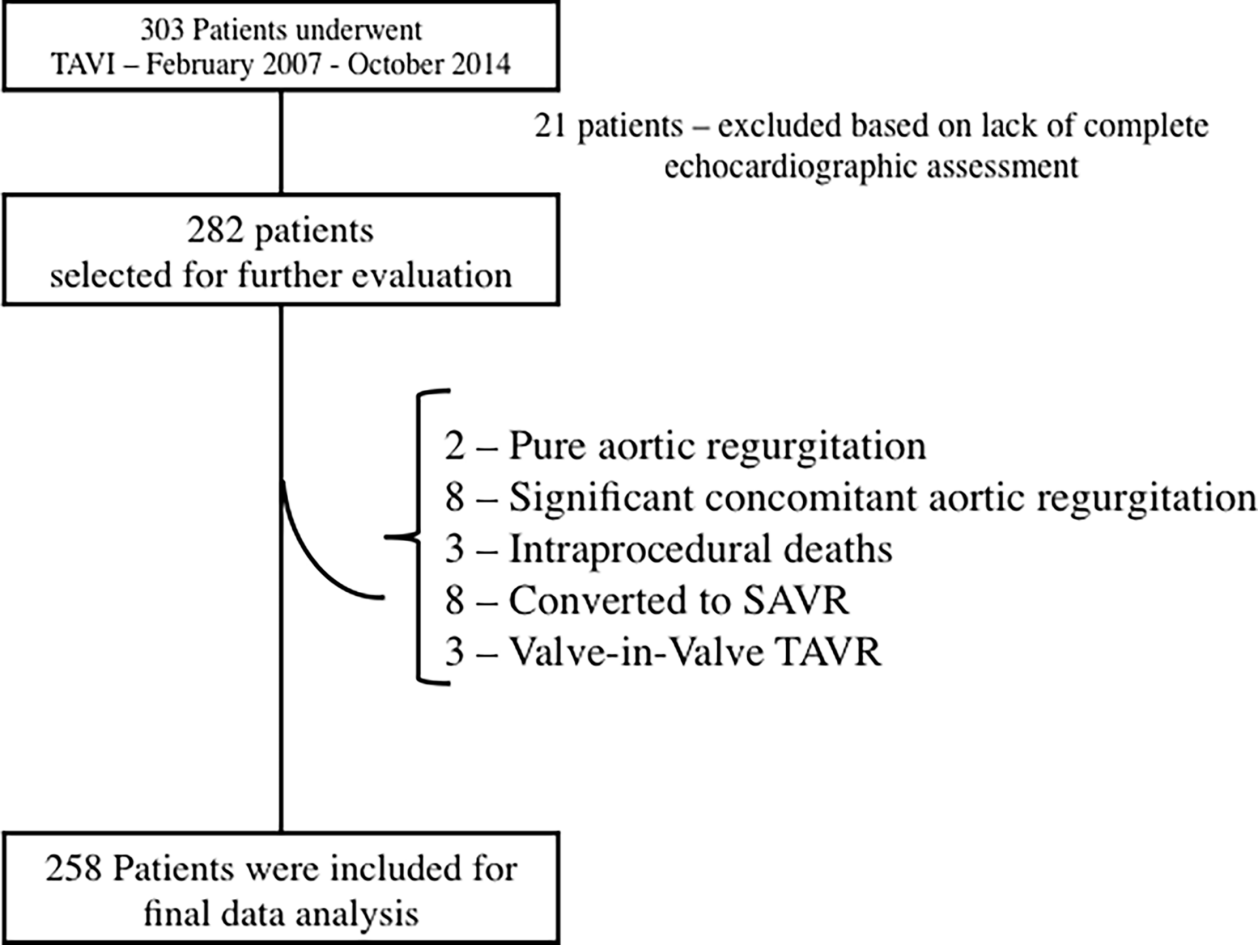
Study protocol of inclusion and exclusion criteria. SAVR = surgical aortic valve replacement, TAVR = transcatheter aortic valve replacement.

Of the 303 symptomatic patients who underwent TAVR, 21 patients were excluded because of incomplete echocardiographic data to calculate the indices of AS severity (i.e. no blood pressure measurement at the time of echocardiographic assessment, no measurable LV outflow tract (LVOT) velocity or diameter) “Fig 1”. Of the remaining 282 patients with complete echocardiographic data, 24 patients were excluded because the lesion was predominantly aortic regurgitation (≥3+AR) (n = 10), the patient underwent valve-in-valve TAVR (n = 3), the patient suffered an intraprocedural death (n = 3), or because of intraprocedural conversion to surgical AVR (n = 8). The final cohort consisted of 258 symptomatic patients with hemodynamically severe AS, defined as an AVA≤1.0cm^2^ and/ or indexed AVA<0.6cm^2^/m^2^.

### Echocardiography examination

All patients had a comprehensive baseline 2D and Doppler echocardiogram prior to the TAVR procedure[14]. LVOT diameter was measured in mid-systole in the parasternal long-axis view using the inner-edge to inner-edge technique, immediately adjacent to the aortic leaflet insertion and parallel to the valve plane. LVOT velocity was recorded using pulsed Doppler in the outflow tract just proximal to the aortic valve using an anteriorly angulated apical 4-chamber view. Velocity curves that demonstrate spectral broadening at peak ejection were excluded. Transvalvular velocity was measured with continuous wave Doppler using the window with the highest velocity. The left ventricular ejection fraction was calculated using the biplane Simpson method. All measurements were averaged from at least 3 cardiac cycles in patients with sinus rhythm and at least 5 consecutive cardiac cycles in patients with atrial fibrillation.

#### Doppler-echocardiographic indices of AS severity

Left ventricular outflow tract cross-sectional area (CSA_LVOT_) was calculated from the LVOT diameter using a circular assumption. Stroke volume was calculated as[15]:

SV=VTI(LVOT)xCSA(LVOT)

where SV is stroke volume and VTI_LVOT_ is the LVOT velocity time integral. Stroke volume index (SVI) was calculated by indexing SV to the body surface area.

Peak transvalvular pressure gradient (PG) was calculated using the peak transvalvular velocity (V_max_) and LVOT velocity (V_LVOT_) using the modified Bernoulli equation[14]:

$$PG=4\;({Vmax}^{2}-{V(LVOT)}^{2})$$

MG was obtained by averaging instantaneous pressure gradients over the ejection period.

AVA was calculated using the continuity equation[16]:

AVA=(VTILVOTxCSALVOT)/VTIAS

where VTI_AS_ is transvalvular velocity time integral.

Percent left ventricular stroke work loss (%SWL), the amount of work lost ejecting blood across the stenotic aortic valve as a function of left ventricular work produced, was derived as[5]:

$$\%SW\;L=\left(\frac{MG}{LVPmean}\right)x\;100\%$$

where LVP_mean_ is mean left ventricular systolic pressure, derived by adding the systolic cuff blood pressure (SBP) and MG.

Valvuloarterial impedance (Zva) was calculated as[8]:

$$\frac{Zva=SBP+MG}{SVI}$$

The relative contribution of the valve load as a function of the global left ventricular load, or “Relative Valve Load (*RVL*)” was calculated as:

$$RVL=\frac{MG}{Zva}$$

### TAVR procedure

TAVRs were performed by a heart valve team consisting of an interventional cardiologist, cardiac surgeon and cardiac anesthesiologist. Balloon expandable Edwards Sapien (Edwards Lifesciences, Irvine, CA) and CoreValve (Medtronic, Inc., Minneapolis, MN) prostheses were implanted based on anatomic and procedural considerations. Prosthetic size selection was based on the aortic annulus dimensions obtained by multi-slice computer tomography and/or transesophageal echocardiography. Access site was primarily by transfemoral approach (n = 230). In patients with severe peripheral vascular disease, a transapical (n = 17) or transaortic (n = 11) approach was used. The procedure was performed with the patient under general anesthesia, and guided by transesophageal echocardiography and fluoroscopy. Procedural success was defined as the successful implantation of a functioning aortic prosthesis without intraprocedural mortality.

### Clinical outcome

The primary endpoint was the predictive value of RVL for overall mortality at 1 year. Secondary endpoints were the predictive value of RVL for cardiovascular mortality at one year and the composite endpoint of overall mortality and need for re-hospitalization for heart failure or cardiogenic shock at 1 year.

### Statistical analysis

Statistical analyses were performed using SAS 9.4. Continuous variables are presented as the mean±SD (for normally distributed data) or as the median and interquartile range when the data is not normally distributed. Continuous variables were compared by unpaired Student’s t-tests or the Wilcoxon rank-sum test. Categorical variables are presented as counts and percentages and compared using Chi Square or Fisher’s exact test.

Receiver operator characteristic curves (ROC) were created for the indices of AS severity and hemodynamic parameters and the primary and secondary outcomes. Cut-off values for chi square test comparisons were selected based on values previously reported in the literature to be associated with a good prognosis in medically managed AS patients or a poor prognosis in AS patients undergoing valve replacement: MG<40mmHg[17–20], AVA>0.75cm^2^[21], %SWL≤25%[9], Zva>5mmHg/ml/m^2^[13], and SVI≤35ml/m^2^[18,19,22,23]. For RVL, the cut-off value was selected as the point of combined maximal sensitivity and specificity for the primary outcome of all cause mortality based on Youden index (J), where *J* = Sensitivity + Specificity– 1.

Time to event data was compared using the log rank test and Kaplan-Meier curves were generated. Univariable analysis and multivariable analysis were performed to identify independent determinants of all-cause mortality. The Cox proportional-hazards model was used to adjust for variables found to have a p-value less than 0.1 in the univariable analysis. These included LVEF ≤40%, atrial fibrillation and the Euroscore II. Estimates of the hazard ratios and their 95% confidence intervals were calculated. Two-sided P values <0.05 were considered statistically significant.

## Results

### Baseline characteristics

The baseline demographics and hemodynamic findings in the 258 patients with hemodynamically severe AS, AR≤2+ and successful implantation of the TAVR prosthesis are shown in “Table 1” and “S1 Table”. The average age was 84.4±6.5 years (50% male). MG was 45.3±15.0mmHg and AVA was 0.69 ± 0.17cm^2^. The median Euroscore II was 5.4% (IQR 3.2–9.9%).

**Table 1:** Baseline demographic, hemodynamic and procedural characteristics of study cohort.

	Overall	Patients died during 1^st^ year post-TAVR	Patients survived 1^st^ year post-TAVR
	(N = 258)	(N = 53)	(N = 205)
***Age—Mean (SD)—Years***	84.4 (6.5)	83.6 (7.0)	84.6 (6.4)
***Sex–n (%Male)***	129 (50.0)	28 (52.8)	101 (49.3)
***BMI—Median (IQR 1- IQR 3)–Kg/m***^***2***^	25.9 (22.6–28.8)	23.8 (22.0–27.1)	26.1 (22.9–28.9)
***Active or ex-smoker–n (%)***	63 (24.4)	17 (32.1)	46 (22.4)
***Dyslipidemia–n (%)***	162 (62.8)	33 (62.3)	129 (62.9)
***DM–n (%)***	92 (35.7)	20 (37.7)	72 (35.1)
***Hypertension–n (%)***	178 (69.0)	36 (67.9)	142 (69.3)
***Angina–n (%)***	53 (20.5)	7 (13.2)	46 (22.4)
***Dyspnea–n (%)***	135 (52.3)	24 (45.3)	111 (54.1)
***Syncope–n (%)***	19 (7.4)	3 (5.7)	16 (7.8)
***Coronary artery disease–n (%)***	208 (80.6)	42 (79.2)	166 (81.0)
***Prior CABG–n (%)***	55 (21.3)	13 (24.5)	42 (20.5)
***Prior MVR–n (%)***	4 (1.6)	1 (1.9)	3 (1.5)
***Prior stroke/TIA–n (%)***	40 (15.5)	7 (13.2)	33 (16.1)
***Peripheral vascular disease–n (%)***	46 (17.8)	6 (11.3)	40 (19.5)
***Atrial fibrillation/flutter–n (%)***	98 (38.0)	27 (50.9)	71 (34.6)
***Defibrillator or biventricular pacing–n (%)***	19 (7.4)	4 (7.5)	15 (7.3)
***LVEF ≤40%–n (%)***	57 (22.1)	16 (30.2)	41 (20.0)
***eGFR ≤ 30 ml/min/m***^***2***^***– n (%)***	38 (14.7)	12 (22.6)	26 (12.7)
***Pulmonary hypertension–n (%)***	29 (11.2)	7 (13.2)	22 (10.7)
***COPD–n (%)***	40 (15.5)	13 (24.5)	27 (13.2)
***Cancer–n (%)***	50 (19.4)	11 (20.8)	39 (19.0)
***Euroscore II %—Median (IQR 1—IQR3)***	5.4 (3.2–9.9)	6.8 (4.4–11.8)	4.9 (3.0–9.4)
***SBP mmHg–Mean (SD)***	134.6 (28.5)	132.3 (31.4)	135.7(28.1)
***MG–Mean (SD)***	44.8 (14.9)	38.7 (14.2)	46.4(14.7)
***AVA–cm***^***2***^ ***–Mean (SD)***	0.69 (0.17)	0.69 (0.17)	0.69 (0.17)
***% SWL—%—Mean (SD)***	24.8 (6.7)	22.7 (6.5)	25.3 (6.7)
***Zva mmHg/ ml/m***^***2***^***/min–Median (IQR 1—IQR3)***	4.6 (3.9–5.5)	4.9 (4.1–6.5)	4.5 (3.9–5.3)
***SVI ml/m***^***2***^***/min–Mean (SD)***	40.2 (12.4)	34.2 (10.1)	41.1 (12.8)
***LF–n (%)***	100 (38.8)	31 (59.6)	69 (33.7)
***NFLG–n (%)***	37 (14.3)	6 (11.3)	31 (15.1)
***PLFLG–n (%)***	24 (9.3)	9 (17.0)	15 (7.3)
***CLFLG–n (%)***	30 (11.6)	11 (20.8)	19 (9.2)
***RVL ml/m***^***2***^ ***–Median (IQR 1—IQR3)***	9.5 (7.3–12.2)	7.5 (5.4–9.8)	10.0 (8.0–12.6)

AVA: Aortic Valve Area; BMI: Body Mass Index; CABG: Coronary Artery Bypass Graft; CLFLG: Classical low flow low gradient (SVI ≤35 ml/m2/min, MG ≤ 40 and LVEF <50%); COPD: Chronic Obstructive Pulmonary Disease; DM: Diabetes Mellitus; eGFR: Estimated Glomerular Filtration Rate; LF: Low Flow (SVI ≤35 ml/m2/min); LVEF: Left Ventricular Ejection Fraction; MG: Mean Pressure Gradient; MVR: Mitral Valve Replacement; NFLG: Normal flow low gradient (SVI >35 ml/m2/min, MG ≤ 40); PLFLG: paradoxical low flow low gradient (SVI ≤35 ml/m2/min, MG ≤ 40 and LVEF ≥ 50%); RVL: Relavtive Valve Load; SVI: Stroke Volume Index; TIA: Transient Ischemic Attack; Yrs: Years % SWL: Stroke Work Loss.

### All cause mortality at 1-year post-TAVR

Fifty-three patients (20.5%) died at 1-year post-TAVR. Of the 53 deaths, 26 (49.1%) had a cardiovascular cause, 22 (41.5%) had a non-cardiac cause, and in 5 (9.4%) the cause of death could not be defined. The clinical and echocardiographic characteristic of the patients who died and survived at 1-year post-TAVR is summarized in “Table 1”.

On ROC analysis, RVL had the largest area under the curve (AUC = 69.3%) for the prediction of all cause mortality at 1-year post-TAVR “Table 2”. The AUC for RVL was significantly greater than that observed for AVA (p = 0.003), %SWL (p = 0.008) and Zva (p = 0.032), but not statistically larger than MG (p = 0.165) or SVI (p = 0.32) “Table 2”. The best cut-off value for predicting all cause mortality at 1-year post-TAVR was an RVL≤7.95ml/m^2^, which provided a sensitivity and specificity of 60.4% and 75.1%, respectively “Table 3”. The specificity of RVL≤7.95ml/m^2^ was significantly larger than obtained using MG<40mmHg (p = 0.002), AVA>0.75cm^2^ (p = 0.008), %SWL≤25% (p<0.001) Zva≥5mmHg/ml/m^2^ (p = 0.004), and SVI≤35ml/m^2^ (p = 0.045), despite similar or greater sensitivity “Table 3”. Univariate analysis of clinical and hemodynamic prognostic variables in patients with RVL≤7.95ml/m^2^ and >7.95ml/m^2^ are summarized in “Table 4”. Patients with RVL≤7.95ml/m^2^ had a higher prevalence of atrial fibrillation and LVEF≤40%, and a higher Euroscore II compared to patients with RVL>7.95ml/m^2^. When adjusting for these variables in Cox proportional hazard model, RVL continued to show statistical significance as a predictor of all cause mortality 1-year post-TAVR (HR 3.2, CI 1.8–5.9; p< 0.0001).

**Table 2:** Receiver Operator Curve Analysis of the hemodynamic indices for predicting all cause mortality 1-year post-TAVR.

Hemodynamic Index	AUC (%)	*P-value compared to RVL*
***RVL***	69.3 (61.5–77.2)	
***MG***	64.8 (56.0–73.4)	0.1649
***AVA***	51.7 (42.7–60.7)	0.0029*
***% SWL***	60.0 (51.4–68.6)	0.0076*
***Zva***	60.3 (51.5–69.2)	0.0320*
***SVI***	68.0 (60.1–75.9)	0.6465

AUC: Area under the curve; AVA: Aortic valve area; MG: Mean pressure gradient; RVL: Relative valve load; SVI: Stroke volume index; Zva: Valvuloarterial impedance; %SWL: Percent stroke work loss.

* = statistically significant at p <0.05

**Table 3:** Comparison of the sensitivity and specificity of hemodynamic indices for predicting all cause mortality 1-year post-TAVR.

Hemodynamic Index	Sensitivity	*P value* *compared to RVL*	Specificity	*P value* *compared to RVL*
***RVL ≤ 7*.*95ml/m^2^***	60.4%		75.1%	
***MG < 40mmHg***	54.7%	0.508	64.4%	0.0015 *
***AVA*** *>* ***0*.*75 cm***^***2***^	39.6%	0.0347*	62.4%	0.0080 *
***% SWL*** *≤* ***25%***	62.3%	1.000	47.8%	<0.0001*
***Zva ≥ 5mmHg/ml/m***^***2***^	47.2%	0.167	65.4%	0.0039 *
***SVI*** *≤****35ml/m***^***2***^	56.6%	0.774	69.3%	0.0455*

AVA: Aortic valve area; MG: Mean pressure gradient; RVL: Relative valve load; SVI: Stroke volume index; Zva: Valvuloarterial impedance; %SWL: Percent stroke work loss.

* = statistically significant at p <0.05

**Table 4:** Comparison of clinical and hemodynamic variables in patients with RVL >7.95ml/m^2^ and *RVL ≤ 7*.*95ml/m*^*2*^.

* Variables*	*RVL > 7*.*95ml/m*^*2*^ *(N = 175)*	*RVL ≤ 7*.*95ml/m*^*2*^ *(N = 83)*	*P value*
***LVEF ≤40%*, *n (%)***	24 (13.7)	33 (39.8)	<0.0001*
***CAD*, *n (%)***	136 (78.2)	72 (86.8)	0.1013
***eGFR ≤ 30 ml/min/m^2^*, *n (%)***	26 (14.9)	12(14.5)	0.9326
***Sex- male*, *n (%)***	84 (48.0)	45 (54.2)	0.3509
***DM*, *n (%)***	61 (34.9)	30 (36.1)	0.8398
***Hypertension*, *n (%)***	124 (70.9)	58 (69.9)	0.8722
***AR–PVL ≥ 2+*, *n (%)***	25 (14.3)	11 (13.3)	0.8231
***AR—PVL≥3+*, *n (%)***	6 (3.4)	2 (2.4)	** 1.0000
***Atrial fibrillation/flutter*, *n (%)***	54 (30.9)	48 (57.8)	<0.0001*
***Ever smoked*, *n (%)***	66 (37.7)	36 (43.4)	0.3851
***Euroscore II (%) Median (IQR1-3)***	4.8 (3.0–9.3)	5.8 (3.6–12.1)	0.0296*
***Age (yrs)–Mean (± SD)***	84.4 ± 6.8	84.4 ± 6.1	0.9879
***BMI (Kg/m***^***2***^***)–Mean (± SD)***	25.8± 5.6	26.2± 4.4	0.5724

AR–PVL: Aortic regurigration with paravalvular leak; CAD: Coronary artery disease; DM: Diabetes mellitus; eGFR: Estimated glomerular filtration rate; LVEF: Left ventricular ejection fraction; RVL: Relative valve load; Yrs: Years

* = statistically significant at p <0.1

Wilcoxon rank-sum test was used for the Euroscore II p-value since the Euroscore II variable is skewed.

**Fisher exact test P-value

**Fig 2:**
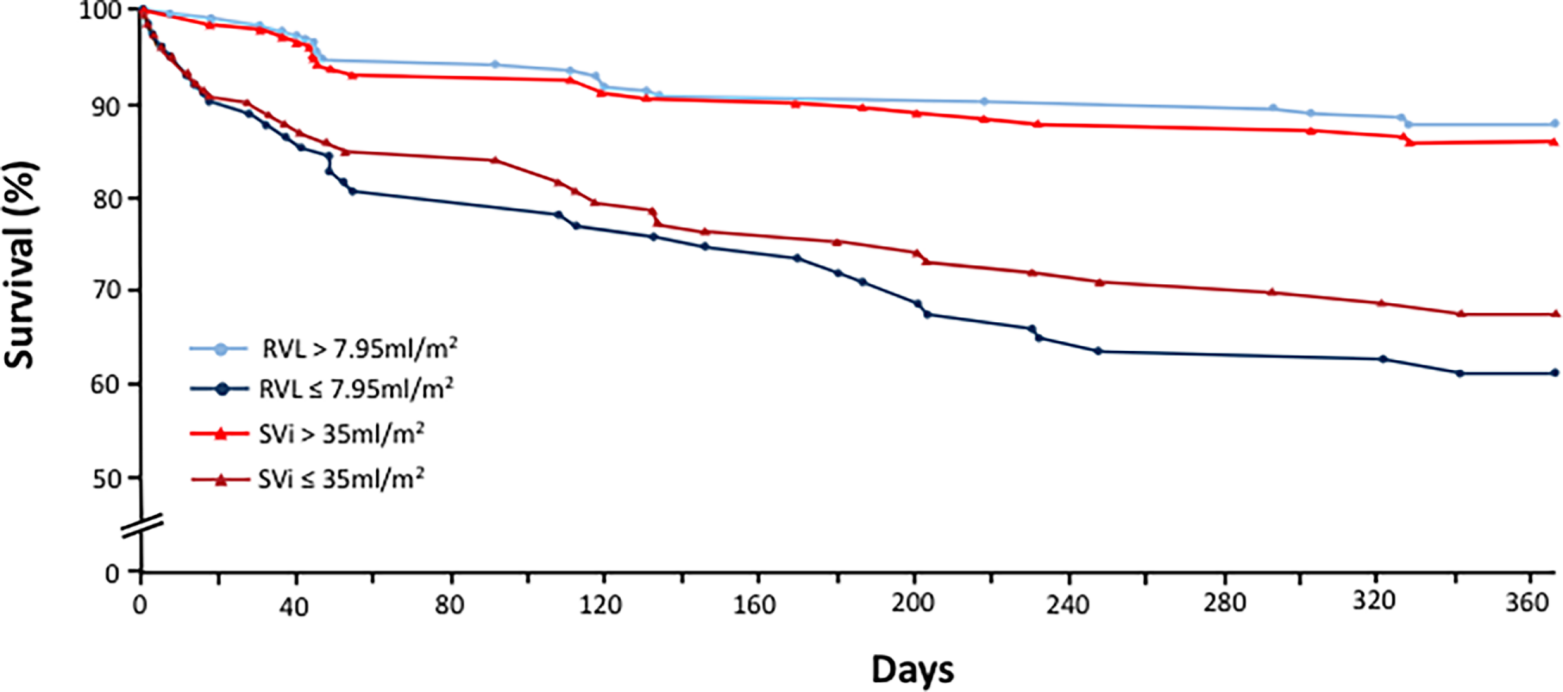
Kaplan-Meier survival curves post-TAVR based on RVL and SVI. RVL = Relative Valve Load, SVI = stroke volume index.

Kaplan-Meier survival curves for the two most specific indices (RVL and SVI) for predicting all cause mortality are shown in “Fig 2”. Survival at 1-year post-TAVR was 88.0% for patients with RVL>7.95ml/m^2^ and 61.4% for patients with RVL≤7.95ml/m^2^ (27% margin of difference, p<0.0001). Using an SVI cut-point of 35ml/m^2^, the margin of difference for all cause mortality at 1-year post-TAVR was smaller at 18% (p = 0.0005).

### Cardiovascular mortality 1-year post-TAVR

Cardiovascular death occurred in 26 patients 1-year post-TAVR. RVL had the largest AUC for predicting cardiovascular mortality 1-year post-TAVR (AUC = 75.0%) “Table 5”. The AUC for RVL was significantly larger than that for AVA (p<0.001) and %SWL (p = 0.004), but not statistically larger than the AUC for MG (p = 0.058), Zva (p = 0.22) or SVI (p = 0.78). RVL≤7.95ml/m^2^ had a sensitivity of 69.2% and specificity of 72.3% for the prediction of cardiovascular death 1-year post-TAVR “Table 6”. The specificity of RVL≤7.95ml/m^2^ was larger than that achieved with MG≤40mmHg (p = 0.007), AVA≥0.75cm^2^ (p = 0.028), %SWL≤25% (p<0.001) and Zva>5mmHg/ml/m^2^ (p = 0.020), but not statistically superior to SVI<35ml/m^2^ (p = 0.105).

**Table 5:** Receiver operator curve analysis of the hemodynamic indices for predicting cardiovascular mortality 1-year post-TAVR.

Hemodynamic Index	AUC (%)	*P-value compared to RVL*
***RVL***	75.0 (66.9–84.1)	
***MG***	67.2 (54.2–80.2)	0.0580
***AVA***	53.8 (41.9–65.7)	0.0003*
***% SWL***	63.3 (50.3–76.2)	0.0044*
***Zva***	66.9 (55.8–78.0)	0.2229
***SVI***	73.6 (64.9–82.3)	0.7777

AUC: Area under the curve; AVA: Aortic valve area; MG: Mean pressure gradient; RVL: Relative valve load; SVI: Stroke volume index; Zva: Valvuloarterial impedance; %SWL: Percent stroke work loss.

* = statistically significant at p <0.05

**Table 6:** Comparison of the sensitivity and specificity of hemodynamic indices for predicting cardiovascular mortality 1-year post-TAVR.

Hemodynamic Index	Sensitivity	*P value* *compared to RVL*	Specificity	*P value* *compared to RVL*
***RVL ≤ 7*.*95ml/m***^***2***^	69.2%		72.3%	
***MG < 40mmHg***	61.5%	0.5000	63.4%	0.0065 *
***AVA > 0*.*75 cm***^***2***^	38.5%	0.0078 *	62.1%	0.0276 *
***SWL ≤ 25%***	61.5%	0.5000	47.6%	<0.0001*
***Zva ≥ 5mmHg/ml/m***^***2***^	57.7%	0.5811	64.8%	0.0195*
***SVI ≤35ml/m***^***2***^	69.2%	1.000	67.8%	0.1048

AVA: Aortic valve area; MG: Mean pressure gradient; RVL: Relative valve load; SVI: Stroke volume index; Zva: Valvuloarterial impedance; %SWL: Percent stroke work loss.

* = statistically significant at p <0.05

### All cause mortality or cardiovascular re-admission at 1 year

At 1-year post-TAVR, 82 patients either died or required re-hospitalization for heart failure or cardiogenic shock. RVL had an AUC of 61.7% “Table 7”. RVL≤7.95 ml/m^2^ had a sensitivity of 43.8% and a specificity of 73.3% for the combined endpoint 1-year post-TAVR “Table 8”. The specificity was larger than that observed for MG<40mmHg (p = 0.033), %SWL≤25% (p<0.001) and Zva≥5mmHg/ml/m^2^ (p = 0.019), but not statistically superior to AVA >0.75cm^2^ (p<0.090) or SVI ≤ 35ml/m^2^ (p = 0.35).

**Table 7:** Receiver operator curve analysis of the hemodynamic indices for predicting the combined outcome of all cause mortality or cardiovascular re-admission 1-year post-TAVR.

Hemodynamic Index	AUC (%)	*P-value compared to RVL*
***RVL***	61.7 (54.3–69.0)	
***MG***	61.0 (53.4–68.6)	0.8139
***AVA***	57.1 (49.5–64.7)	0.3962
***% SWL***	56.4 (48.6–64.2)	0.0968
***Zva***	54.6 (46.8–62.4)	0.0473*
***SVI***	61.0 (53.2–68.5)	0.8161

AUC: Area under the curve; AVA: Aortic valve area; MG: Mean pressure gradient; RVL: Relative valve load; SVI: Stroke volume index; Zva: Valvuloarterial impedance; %SWL: Percent stroke work loss.

* = statistically significant at p <0.05

**Table 8:** Comparison of the sensitivity and specificity of hemodynamic indices for predicting the combined outcome of all cause mortality or cardiovascular re-admission 1-year post-TAVR.

Hemodynamic Index	Sensitivity	*P value* *compared to RVL*	Specificity	*P value* *compared to RVL*
***RVL ≤ 7*.*95ml/m***^***2***^	43.7%		73.3%	
***MG < 40mmHg***	48.8%	0.3458	65.7%	0. 0326*
***AVA > 0*.*75 cm***^***2***^	42.5%	0.869	64.5%	0.09
***SWL ≤ 25%***	60.0%	0.0093	48.8%	<0.0001*
***Zva ≥ 5mmHg/ml/m***^***2***^	41.3%	0.6831	64.5%	0. 0191*
***SVI ≤35ml/m***^***2***^	47.5%	0.4669	70.4%	0. 3532

AVA: Aortic valve area; MG: Mean pressure gradient; RVL: Relative valve load; SVI: Stroke volume index; Zva: Valvuloarterial impedance; %SWL: Percent stroke work loss.

* = statistically significant at p <0.05

### Predictive value of RVL for all cause mortality 1-year post-TAVR in normal flow and low flow severe AS

Normal flow AS (SVI>35 ml/m^2^) was present in 158 patients and low flow AS (SVI≤35 ml/m^2^) in 100 patients. At 1-year post-TAVR, all cause mortality occurred in 22 patients with normal flow AS (13.9%) and 31 patients with low flow AS (31.0%). In patients with normal flow AS, all cause mortality 1-year post-TAVR was 36.8% in patients with RVL≤7.95ml/m^2^ and 10.96% in patients with RVL>7.95ml/m^2^ (Chi square value 9.39, LR 7.36, p = 0.0068). In patients with low flow AS, all cause mortality 1-year post-TAVR was 39.1% in patients with an RVL≤7.95ml/m^2^ and 17.4% in patients with an RVL>7.95ml/m^2^ (Chi square value 4.35, LR 4.66, p = 0.03).

### Predictive value of RVL for all cause mortality 1-year post-TAVR in low gradient severe AS

Low gradient (MG<40 mmHg) severe AS was present in 101 patients (classical low flow low gradient AS [LVEF<50%] = 30, paradoxical low flow low gradient AS [LVEF≥50%] = 23, normal flow low gradient AS = 48). At 1-year post-TAVR, 29 patients (28.7%) with low gradient AS had died, 41.9% (26 of 62 patients) with RVL≤7.95ml/m^2^ and 7.7% (3 of 39 patients) with RVL>7.95ml/m^2^ (Chi square value 13.72, p = 0.0002). All cause mortality 1-year post-TAVR with an RVL≤7.95ml/m^2^ compared to an RVL>7.95ml/m^2^ was 42.3% vs. 0% in classical low flow low gradient AS (p = 0.10), 33.3% vs. 50.0% in paradoxical low flow low gradient AS (p = 0.64), and 53.3% vs. 6.1% in normal flow low gradient AS (p = 0.0002), respectively. Only two patients with paradoxical low flow low gradient AS had RVL>7.95ml/m^2^.

## Discussion

The main finding of this study is that the novel hemodynamic index, RVL, can be used to predict the outcome of AS patients following TAVR. Pre-procedural RVL was a strong predictor of all cause mortality, cardiovascular mortality and the combined outcome of all cause mortality and re-hospitalization for heart failure or cardiogenic shock 1-year post-TAVR. RVL≤7.95ml/m^2^ provided a better prediction of all cause mortality and cardiovascular mortality at 1 year with an improved specificity compared to that obtained with conventional hemodynamic indices of AS severity, MG and AVA, as well as other proposed hemodynamic indices such as Zva and %SWL. Furthermore, RVL maintained its predictive value in patients with both normal and low flow severe AS.

Progressive left ventricular pressure overload, whether from a valvular load, arterial load or combined process, will eventually lead to left ventricular failure[10]. Zva provides an approximation of the global LV afterload and has been shown to be associated with the presence of LV systolic and diastolic dysfunction, as well as clinical outcomes in patients with moderate and severe AS[8,11]. However, TAVR only relieves the valvular load, and the benefits of a TAVR may be limited in the AS patient in whom a high LV afterload relates predominantly to an arterial load[13]. In addition, the hemodynamic indices used to determine AS severity are influenced by the arterial load, potentially affecting the conclusion as to the severity of the valve stenosis and the benefits of TAVR[6,24]. An increase in arterial load can result in the stenosis appearing less severe when evaluated by MG, but more severe when evaluated by AVA[6,24]. If AVA is given preference during the assessment, the presumed benefits of TAVR may be overestimated. In contrast, preference to MG can lead to a potential underestimation of the benefits.

RVL provides a measure of the valvular load as a relative proportion of the global LV afterload. Thus, patients with a higher RVL would be expected to realize a greater benefit from TAVR in comparison to those with a smaller RVL in whom the valve has a relatively smaller contribution to the global LV load. In this study, RVL≤7.95ml/m^2^ was able to predict an adverse outcome following TAVR, providing a better specificity for overall mortality and cardiovascular mortality at one year compared to MG<40mmHg, AVA>0.75cm^2^, %SWL≤25% and Zva≥5mmHg/ml/m^2^, with an equivalent or better sensitivity. In this regard, RVL appears to be a useful measure to help identify which patients may fail to benefit from TAVR.

An important finding of our study is the confirmation of the strong predictive value of SVI on 1-year all cause mortality and cardiovascular mortality following TAVR. SVI has been shown to be an important predictor of survival in patients with native AS, and more recently, in AS patients undergoing surgical AVR and TAVR[18,19,22,23,25,26]. The presence of low flow, defined as a SVI<35ml/m^2^, is associated with worse outcomes[18–20,22,23,26]. Thus, SVI should be strongly considered when evaluating a patient for TAVR. However, while a reduced SVI may be prognostically important, it is a measure of transvalvular volume flow that in isolation does not provide information on the hemodynamic severity of the valve stenosis, may not be caused by severe AS, but rather, may be a manifestation of a coexisting condition (i.e. coronary artery disease, hypertension, etc). In contrast, RVL provides information on the severity of the valve stenosis (relative contribution of the valve load to the total LV load), as well as the prognosis following valve intervention, potentially providing a better index for deciding on the benefits of valve intervention. Importantly, we observed that RVL≤7.95ml/m^2^ provided a higher specificity for all cause mortality at 1-year post-TAVR, and a trend for a better specificity for cardiovascular mortality (p = 0.1), compared to SVI≤35 ml/m^2^. Further studies are warranted in a larger population to confirm the incremental benefit of RVL beyond SVI.

Patients with low flow AS represent a challenging subset to manage as there is often uncertainty as to the benefits of valve intervention[27]. In low flow AS, the “true” severity of the valve stenosis is often unclear, the prognosis of patients is generally worse than normal flow AS, and valve intervention is associated with higher risks[7,18,19,22,27]. Clinicians may underestimate the benefits of TAVR because the MG is small, or alternatively, overestimate the benefits because the small AVA relates to the phenomena of pseudo-severe AS. Zva is flow-dependent and cannot distinguish between true and pseudo-severe AS, or the extent to which the valve accounts for the global LV load[28]. %SWL is also strongly dependent on flow and may underestimate AS severity under low flow conditions[5]. An important finding of our study is that RVL provided a robust predictor of all cause mortality at 1-year post-TAVR in the subgroup of patients with both normal and low flow AS.

### Limitations

This is a single center study and the number of events at 1 year (53 deaths) limits our ability to identify potential interactions and associations of RVL with other factors affecting mortality. While the results are promising, this index should be validated in a larger TAVR population before we can recommend its incorporation into clinical decision-making.

Frailty is an important predictor of outcome following TAVR, but was not objectively measured in this population. We cannot exclude the possibility of an association between frailty and RVL in our study cohort.

Data on valve efficacy at 1 year was not available to determine the relative impact of valve device performance on patient outcome. However, only 7 patients had >2+AR after valve implantation and data from multicenter clinical trials suggest an excellent valve durability at one year[29,30].

## Conclusion

In AS patients undergoing TAVR, the pre-procedural hemodynamic index, RVL, provides a strong predictor of all cause mortality and cardiovascular mortality at 1-year post-TAVR. RVL≤7.95ml/m^2^ identifies AS patients at increased risk for death 1 year post TAVR and can predict outcome in patients with both normal and low flow AS. If validated in a larger patient population, RVL may provide a useful index for individual clinical decision-making on the benefits of TAVR.

## Supporting information

S1 Table Procedural details and causes of device failure. (DOCX)
