# Supplementary material for: A novel echocardiographic hemodynamic index for predicting outcome of aortic stenosis patients following transcatheter aortic valve replacement
Source: PLoS One. 2018 Apr 26;13(4):e0195641. doi: 10.1371/journal.pone.0195641 (PMC5919479; doi:10.1371/journal.pone.0195641)
Supplement: S1 Table — (DOCX) [file pone.0195641.s001.docx]

| **Supplemental Table 1. Procedural details and causes of device failure.** | | | |
| --- | --- | --- | --- |
|  | **Overall** | **Patients died during 1st year post-TAVR** | **Patients survived 1st year post- TAVR** |
| ***Valve type*** | (N=258) | (N= 53) | (N= 203) |
| ***Balloon Expandable valve – n (%)*** | 57 (22.1) | 9 (17.0) | 48 (23.4) |
| ***Self Expandable valve – n (%)*** | 201 (77.9) | 44 (83.0) | 157 (76.6) |
| ***Access*** |  |  |  |
| ***Femoral – n (%)*** | 230 (89.1) | 48 (90.6) | 182 (88.8) |
| ***Transaortic – n (%)*** | 11 (4.3) | 1 (1.9) | 10 (4.9) |
| ***Transapical – n (%)*** | 17 (6.6) | 4 (7.5) | 13 (6.3) |
| ***Device Success – n (%)*** | 236 (91.5) | 46 (86.8) | 190 (92.7) |
| ***Causes of Device Faliure**** |  |  |  |
| ***AR>2+ – n (%)*** | 6 (2.3) | 2 (3.8) | 4 (2.0) |
| ***Valve Malposition – n (%)*** | 7 (2.7) | 2 (3.8) | 5 (2.4) |
| ***Valve embolization – n (%)*** | 9 (3.5) | 2 (3.8) | 7 (3.4) |
| ***Need of a second valve^$^ – n (%)*** | 4 (1.6) | 2 (3.8) | 2 (1.0) |

* Patients may have more than one reason contributing to device failure.
$Excluding implantation of a second valve for device embolization.
